# Disease burden and trends of malignant neoplasm of bone and articular cartilage in childhood in China, 1990–2021 and comparison with United States and India: findings from the Global Burden of Disease Study 2021

**DOI:** 10.3389/fpubh.2025.1481304

**Published:** 2025-07-03

**Authors:** Ruibo Li, Xingyue Yuan

**Affiliations:** ^1^Department of Orthopaedics, Deyang Peoples' Hospital, Deyang, Sichuan, China; ^2^Department of Pathology, Deyang Peoples' Hospital, Deyang, Sichuan, China

**Keywords:** bone cancer, disease burden, childhood, prevention, China

## Abstract

**Objectives:**

This study aims to analyze the disease burden and trends of malignant neoplasm of bone and articular cartilage (MNBAC) in Chinese children from 1990 to 2021 using data from GBD 2021, and to compare them with the United States and India.

**Methods:**

Data on incidence, prevalence, mortality, and disability-adjusted life years (DALYs) for MNBAC in children aged 0–14 years were extracted from GBD 2021. The joinpoint regression analysis model was employed to assess temporal trends, and the average annual percent change (AAPC) was calculated to summarize the trends over the study period.

**Results:**

Globally, the number of incident childhood cases of MNBAC increased from 9,827 in 1990 to 12,294 in 2021, with an AAPC of 0.38. Similarly, the prevalence of the disease also increased, with an AAPC of 0.39. However, the mortality rate and DALYs rate showed a slight decline, with an AAPC of −0.16 and −0.16 respectively. At the national level, the United States had the highest incidence and prevalence rates, while India had the highest number of incident cases and DALYs. China showed the largest increase in prevalence, with an AAPC of 1.88. India also demonstrated the most significant decline in mortality and DALYs rates. Additionally, the mortality rate and DALYs rate were slightly higher for male children compared to female patients.

**Conclusion:**

Malignant bone and articular cartilage tumors in children remain a significant public health challenge globally. By strengthening monitoring, increasing investment in medical resources, promoting early screening and intervention, and enhancing international cooperation and exchange, we hope to further reduce the burden of childhood bone cancer and improve children's quality of life and health standards.

## Introduction

Childhood malignancies represent a significant challenge in the field of global public health. Childhood cancer ranks as the sixth leading cause of the total cancer burden globally and the ninth leading cause of childhood disease burden worldwide (1). In high-income countries, the overall 5-year survival rate for children with cancer is ~80%, while in low and middle-income countries, this rate is <30% (2). Malignant neoplasms of bone and articular cartilage (MNBAC), commonly known as bone cancer, are relatively rare but highly aggressive cancers that primarily affect children and young adults, accounting for <1% of all malignancies (3). Despite their rarity, these cancers pose a major challenge due to their aggressiveness, complex treatment requirements, and profound impact on patients' quality of life and survival rates (4), with significantly lower survival rates compared to other types of tumors (5). These tumors not only severely affect children's growth, development, and quality of life but also impose a heavy economic and psychological burden on families and society. Therefore, a deep understanding of the epidemiological characteristics, disease burden, and trends of malignant bone and articular cartilage tumors is crucial for developing effective prevention and control strategies.

As one of the most populous countries in the world, the health status of children in China is directly related to the achievement of global child health goals. In recent years, with the improvement of medical and health standards and the continuous investment in medical resources, the diagnosis and treatment of childhood malignancies in China have significantly improved (6). However, research on the specific burden and trends of malignant bone and articular cartilage tumors in children, especially comparative studies with other countries such as the United States (a developed country) and India (a developing country), remains inadequate.

The Global Burden of Disease Study (GBD), as a leading global public health research project, systematically collects and analyzes health data worldwide, providing a strong scientific basis for assessing the burden of diseases and formulating public health policies. In the GBD 2019 and earlier versions of the database, MNBAC was classified as other malignancies. GBD 2021, for the first time, reported on MNBAC, providing valuable data support for a deeper understanding of the prevalence of MNBAC globally and in specific regions.

In light of this, this study aims to analyze the disease burden and trends of malignant bone and articular cartilage tumors in Chinese children from 1990 to 2021 using data from GBD 2021, and to compare them with the United States and India. Through this research, we hope to reveal the effectiveness and gaps in the prevention and control of malignant bone and articular cartilage tumors in children across different countries and regions, providing references for further optimizing resource allocation and enhancing prevention and treatment capabilities.

## Methods

### Data sources

This study utilized data from the GBD 2021, which provides comprehensive estimates of health losses caused by 371 diseases (including MNBAC) and injuries in 204 countries and regions from 1990 to 2021 (7). The introduction and estimation methods of GBD 2021 have been described in detail in previous systematic analytical studies on the global burden of disease (7, 8). For non-fatal estimates, data was sourced from scientific literature, household survey data, epidemiological surveillance data, disease registries, clinical informatics, as well as online research databases, government and international organization websites, searches for published reports, and datasets provided by GBD collaborators. Mortality estimates were primarily derived from life registration, autopsy, surveys, police, or surveillance data from all countries and regions. Consistent disease estimates were obtained using the epidemiological state transition disease modeling software Dismod-MR (developed by the Institute for Health Metrics and Evaluation) and the Bayesian meta-regression software MR-BRT (also developed by the Institute for Health Metrics and Evaluation), with adjustments made for differences in measurement methods and the level of research on case definitions (7, 9). In this investigation, the determinations and their 95% uncertainty interval (UI) for incidence, prevalence, mortality and disability-adjusted life years (DALYs) relating to MNBAC of children (0–14 years) were drawn from the GBD 2021 data.

It should be noted that the GBD 2021 database reports MNBAC as a single aggregated category. This category encompasses multiple subtypes with distinct epidemiological characteristics, biological behaviors, prognoses, and treatment strategies, primarily including osteosarcoma, Ewing sarcoma, and chondrosarcoma. In this study, the data we utilized represents the overall aggregated estimates for this combined category and does not differentiate between specific subtypes. This limitation restricts our ability to analyze the unique burden patterns and trends of individual subtypes within the pediatric population.

All data used in this study were obtained from publicly available, de-identified databases (GBD 2021). No new human studies involving direct recruitment or intervention were conducted. No potentially identifiable images or data are presented in this study.

### Statistics analysis

This study employed a commonly used statistical method in epidemiological research, the joinpoint regression analysis model, to assess the temporal trends of incidence, prevalence, mortality and DALYs rate (10). This model aids in calculating the annual percent change (APC) and its accompanying 95% confidence interval (CI), describing the trend of incidence rates within a specific timeframe. Furthermore, to comprehensively evaluate the observed trends, the average annual percent change (AAPC) was calculated, which incorporates summary trend data from the study period of 1990–2021. The AAPC serves as a summary measure of trends within a prespecified fixed interval, calculated as the weighted average of APCs, enabling us to describe the average APC during the study period with a single number. The value of the AAPC indicates the annual percent change (increase, decrease, or no change). For example, an AAPC of 0.1 implies an annual growth rate increase of 0.1%. When the estimated APC or AAPC value is greater than zero, it indicates an upward trend during the specified time interval. Conversely, when the estimated APC or AAPC value is less than zero, it suggests a downward trend. When the estimated APC or AAPC value is zero, it indicates that the trend remains stable (11, 12).

The incidence rate, prevalence rate, and mortality rate were expressed as predictions per 100,000 population, while DALYs rate was expressed per 100,000 person-years, including their 95% UI. All analyses and graphical representations were conducted using RStudio software (version 4.3.1) and the Joinpoint regression program (version 5.0.2).

## Results

The global number of incident childhood cases increased from 9,827 (95% UI: 7,856–12,123) in 1990 to 12,294 in 2021 (95% UI: 10,174–14,565), while the incidence rate increased from 0.57 per 100,000 population (95% UI: 0.45–0.7) in 1990 to 0.61 per 100,000 population (95% UI: 0.51–0.72) in 2021, with an AAPC of 0.38 (95% CI: 0.33–0.43). In 2021, there were 7,114 male incident cases (95% UI: 5,334–8,855), with the incidence of 0.69 per 100,000 population (95% UI: 0.51–0.85), and 5,181 female incident cases (95% UI: 4,310 to 6,394), with the incidence of 0.53 per 100,000 population (95% UI: 0.44 to 0.66; Table 1).

**Table 1:** The incidence and AAPCs of malignant neoplasm of bone and articular cartilage in childhood from 1990 to 2021.

**Characteristic**		**1990**		**2021**		**AAPCs**
		**Incident cases**	**Incidence (per 100,000 population)**	**Incident cases**	**Incidence (per 100,000 population)**	
Global	Total	9,827 (7,856–12,123)	0.57 (0.45–0.7)	12,295 (10,175–14,566)	0.61 (0.51–0.72)	0.38 (0.33 to 0.43)
	Male	4,819 (3,539–5,782)	0.54 (0.4–0.65)	7,114 (5,334–8,855)	0.69 (0.51–0.85)	0.92 (0.88 to 0.96)
	Female	5,008 (3,579–7,417)	0.59 (0.42–0.88)	5,181 (4,310–6,394)	0.53 (0.44–0.66)	−0.22 (−0.31 to −0.13)
China	Total	1,215 (810–2,186)	0.38 (0.25–0.69)	1,581 (1,100–2,013)	0.61 (0.42–0.78)	1.87 (1.26 to 2.49)
	Male	630 (438–1,015)	0.38 (0.26–0.61)	1,021 (657–1,354)	0.74 (0.47–0.98)	2.57 (1.99 to 3.16)
	Female	585 (344–1,358)	0.38 (0.23–0.89)	559 (342–840)	0.46 (0.28–0.69)	0.93 (0.24 to 1.61)
India	Total	1,598 (961–2,107)	0.49 (0.29–0.65)	1,815 (1,397–2,300)	0.5 (0.38–0.63)	0.13 (−0.03 to 0.29)
	Male	796 (412–1,164)	0.47 (0.24–0.68)	1,090 (765–1,458)	0.57 (0.4–0.76)	0.97 (0.79 to 1.16)
	Female	802 (397–1,279)	0.51 (0.25–0.82)	726 (491–1,006)	0.42 (0.28–0.58)	−0.88 (−1.07 to −0.69)
United States	Total	341 (322–359)	0.61 (0.58–0.64)	384 (351–415)	0.65 (0.59–0.7)	0.06 (−0.14 to 0.26)
	Male	166 (155–176)	0.58 (0.54–0.62)	213 (190–235)	0.7 (0.63–0.77)	0.23 (−0.09 to 0.54)
	Female	175 (165–186)	0.64 (0.6–0.68)	171 (158–184)	0.59 (0.54–0.63)	−0.13 (−0.31 to 0.06)

AAPCs, average annual percent changes.

**Figure 1:**
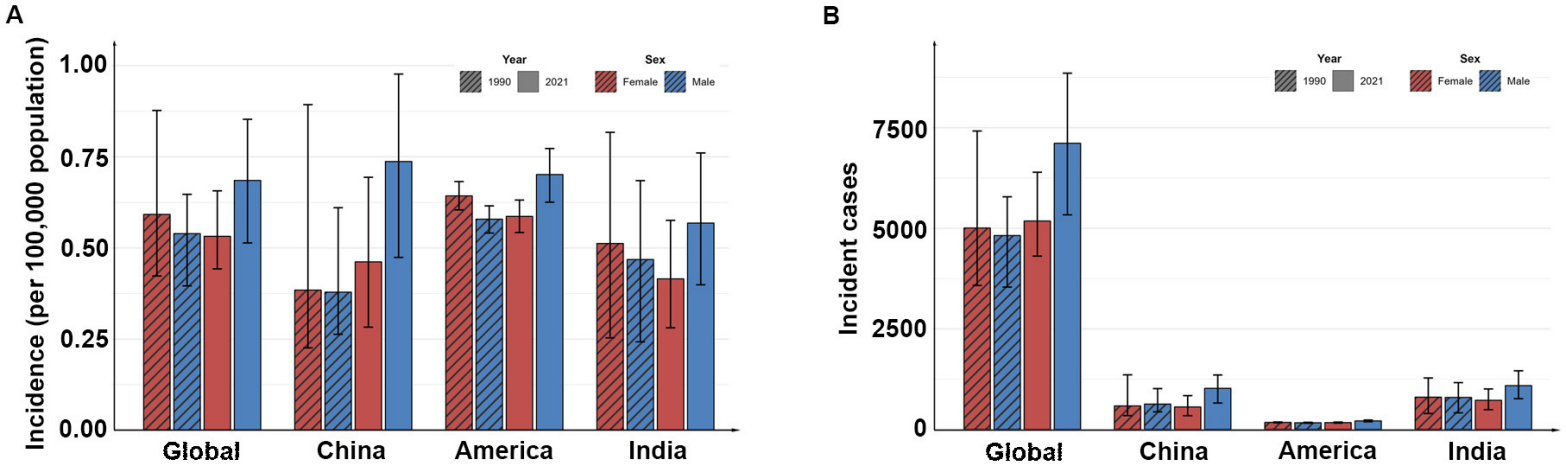
The incidence **(A)** and the incident cases **(B)** in different countries in 1990 and 2021.

In 1990, the number of incident childhood cases in India was ~1,598 (95% UI: 961 to 2,107), higher than that of China, which was 1,215 (95% UI: 810 to 2,186), and the United States, which was 341 (95% UI: 322–359). However, the country with the highest incidence rate in 1990 was the United States, at 0.61 per 100,000 population (95% UI: 0.58–0.64). By 2021, the number of incident childhood cases in all three countries had increased, with India still having the highest number of children cases, reaching 1,815 (95% UI: 1,397–2,300). Furthermore, from 1990 to 2021, the incidence rate in all three countries showed an upward trend, with the most significant increase observed in China. The incidence rate in China rose from 0.38 per 100,000 population (95% UI: 0.25–0.69) in 1990–0.61 per 100,000 population (95% UI: 0.42–0.78) in 2021, with an average annual increase of 1.87% (95% CI: 1.26%−2.49%; Table 1, Figure 1).

**Figure 2:**
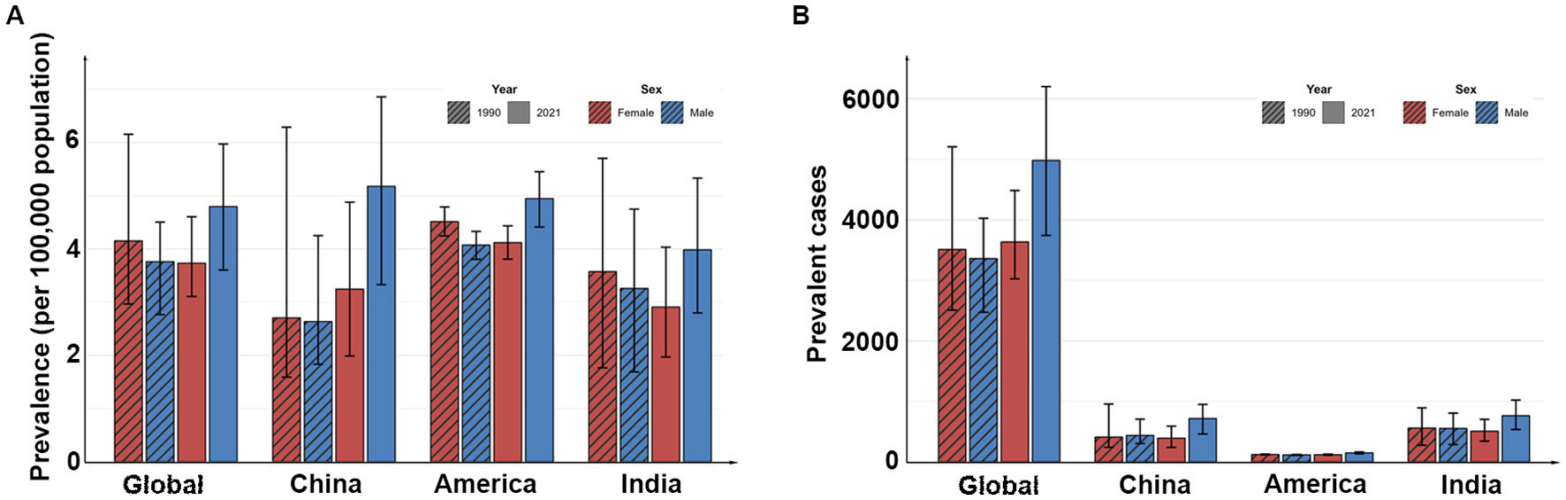
The prevalence **(A)** and the prevalent cases **(B)** in different countries in 1990 and 2021.

The global number of prevalent childhood cases in 2021 was 86,160 (95% UI: 71,359–102,052), up from 68,712 (95% UI: 54,934–84,901) in 1990, and the prevalence also increased, from 3.95 per 100,000 population (95% UI: 3.16–4.88) in 1990 to 4.28 per 100,000 population (95% UI: 3.55–5.07) in 2021, an average annual increase of 0.39% (95% CI: 0.34%−0.44%). At the national level, the number and prevalence of the disease also increased in China, India and the United States from 1990 to 2021. India had the highest number of children cases, with 11,136 (95% UI: 6,696–14,692) in 1990 and 12,721 (95% UI: 9,797–16,099) in 2021, while the United States had the highest prevalence, with 4.29 per 100,000 population (95% UI: 4.05–4.52) in 1990 and 4.54 per 100,000 population (95% UI: 4.15–4.92) in 2021. In addition, China had the largest increase in prevalence, with an average annual increase of 1.88% (95% CI: 1.27%−2.5%; Table 2, Figure 2).

**Table 2:** The prevalence and AAPCs of malignant neoplasm of bone and articular cartilage in childhood from 1990 to 2021.

**Characteristic**		**1990**		**2021**		**AAPCs**
		**Prevalent cases**	**Rate (per 100,000 population)**	**Prevalent cases**	**Prevalence (per 100,000 population)**	
Global	Total	68,712 (54,934–84,901)	3.95 (3.16–4.88)	86,160 (71,359–102,052)	4.28 (3.55–5.07)	0.39 (0.34 to 0.44)
	Male	33,599 (24,724–40,254)	3.76 (2.77–4.51)	49,808 (37,418–62,013)	4.8 (3.6–5.97)	0.93 (0.89 to 0.97)
	Female	35,113 (25,084–52,062)	4.15 (2.97–6.16)	36,351 (30,248–44,845)	3.73 (3.11–4.61)	−0.22 (−0.3 to −0.13)
China	Total	8,499 (5,665–15,331)	2.67 (1.78–4.82)	11,104 (7,723–14,152)	4.28 (2.97–5.45)	1.88 (1.27 to 2.5)
	Male	4,384 (3,049–7,069)	2.64 (1.83–4.25)	7,174 (4,614–9,505)	5.18 (3.33–6.86)	2.6 (2.01 to 3.18)
	Female	4,115 (2,420–9,564)	2.71 (1.59–6.29)	3,930 (2,405–5,908)	3.25 (1.99–4.88)	0.93 (0.24 to 1.62)
India	Total	11,136 (6,696–14,692)	3.41 (2.05–4.5)	12,721 (9,797–16,099)	3.47 (2.67–4.39)	0.15 (0 to 0.31)
	Male	5,538 (2,870–8,070)	3.26 (1.69–4.75)	7,640 (5,365–10,221)	3.99 (2.8–5.33)	1 (0.82 to 1.19)
	Female	5,598 (2,766–8,930)	3.58 (1.77–5.7)	5,081 (3,440–7,046)	2.91 (1.97–4.03)	−0.86 (−1.06 to −0.67)
United States	Total	2,398 (2,266–2,526)	4.29 (4.05–4.52)	2,700 (2,469–2,923)	4.54 (4.15–4.92)	0.06 (−0.13 to 0.26)
	Male	1,167 (1,090–1,240)	4.07 (3.81–4.33)	1,503 (1,340–1,656)	4.95 (4.41–5.45)	0.23 (−0.08 to 0.55)
	Female	1,231 (1,158–1,307)	4.51 (4.24–4.79)	1,197 (1,107–1,289)	4.12 (3.81–4.43)	−0.12 (−0.31 to 0.06)

AAPCs, average annual percent changes.

**Figure 3:**
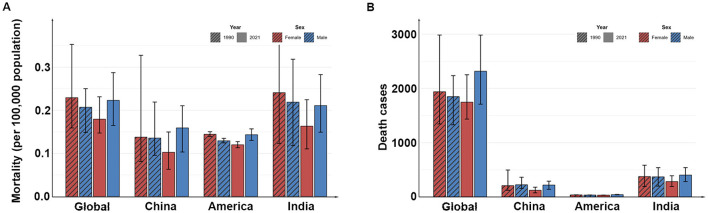
The mortality **(A)** and the death cases **(B)** in different countries in 1990 and 2021.

The number of global child deaths in 2021 was 4,064 (95% UI: 3,419–4,872), up from 3,789 (95% UI: 2,948–4,687) in 1990, but the overall mortality rate declined slightly, from 0.22 per 100,000 population (95% UI: 0.17–0.27) decreased to 0.2 per 100,000 population (95% UI: 0.17–0.24) in 2021, representing an annual decrease of 0.16% (95% CI: 0.09%−0.24%). At the national level, from 1990 to 2021, the number of child deaths decreased in China and India, the number of deaths in the United States remained almost unchanged, the mortality rate in India declined from 0.23 per 100,000 population (95% UI: 0.14–0.3) in 1990 to 0.19 per 100,000 population (95%UI: 0.15–0.24) in 2021, an average annual decline of 0.63% (95% CI: 0.48%−0.78%), while the mortality rate in China and the United States decreased slightly or remained almost unchanged (Table 3, Figure 3).

**Table 3:** The mortality and AAPCs of malignant neoplasm of bone and articular cartilage in childhood from 1990 to 2021.

**Characteristic**		**1990**		**2021**		**AAPCs**
		**Death cases**	**Mortality rate (per 100,000 population)**	**Death cases**	**Mortality rate (per 100,000 population)**	
Global	Total	3,789 (2,948–4,687)	0.22 (0.17–0.27)	4,064 (3,419–4,872)	0.2 (0.17–0.24)	−0.16 (−0.24 to −0.09)
	Male	1,850 (1,329–2,235)	0.21 (0.15–0.25)	2,317 (1,710–2,983)	0.22 (0.16–0.29)	0.35 (0.29 to 0.4)
	Female	1,939 (1,345–2,984)	0.23 (0.16–0.35)	1,747 (1,435–2,252)	0.18 (0.15–0.23)	−0.73 (−0.84 to −0.62)
China	Total	435 (290–800)	0.14 (0.09–0.25)	345 (240–435)	0.13 (0.09–0.17)	0.05 (−0.6 to 0.71)
	Male	226 (159–365)	0.14 (0.1–0.22)	221 (143–292)	0.16 (0.1–0.21)	0.7 (0.07 to 1.33)
	Female	210 (124–498)	0.14 (0.08–0.33)	124 (77–181)	0.1 (0.06–0.15)	−0.82 (−1.53 to −0.1)
India	Total	749 (457–989)	0.23 (0.14–0.3)	690 (536–882)	0.19 (0.15–0.24)	−0.63 (−0.78 to −0.48)
	Male	372 (201–541)	0.22 (0.12–0.32)	405 (286–542)	0.21 (0.15–0.28)	0.16 (0.02 to 0.31)
	Female	377 (193–587)	0.24 (0.12–0.37)	285 (193–392)	0.16 (0.11–0.22)	−1.56 (−1.78 to −1.34)
United States	Total	76 (74–79)	0.14 (0.13–0.14)	78 (73–84)	0.13 (0.12–0.14)	−0.25 (−0.48 to −0.03)
	Male	37 (36–39)	0.13 (0.12–0.14)	44 (40–48)	0.14 (0.13–0.16)	−0.08 (−0.43 to 0.26)
	Female	39 (38–41)	0.14 (0.14–0.15)	35 (33–37)	0.12 (0.11–0.13)	−0.44 (−0.64 to −0.25)

AAPCs, average annual percent changes.

**Figure 4:**
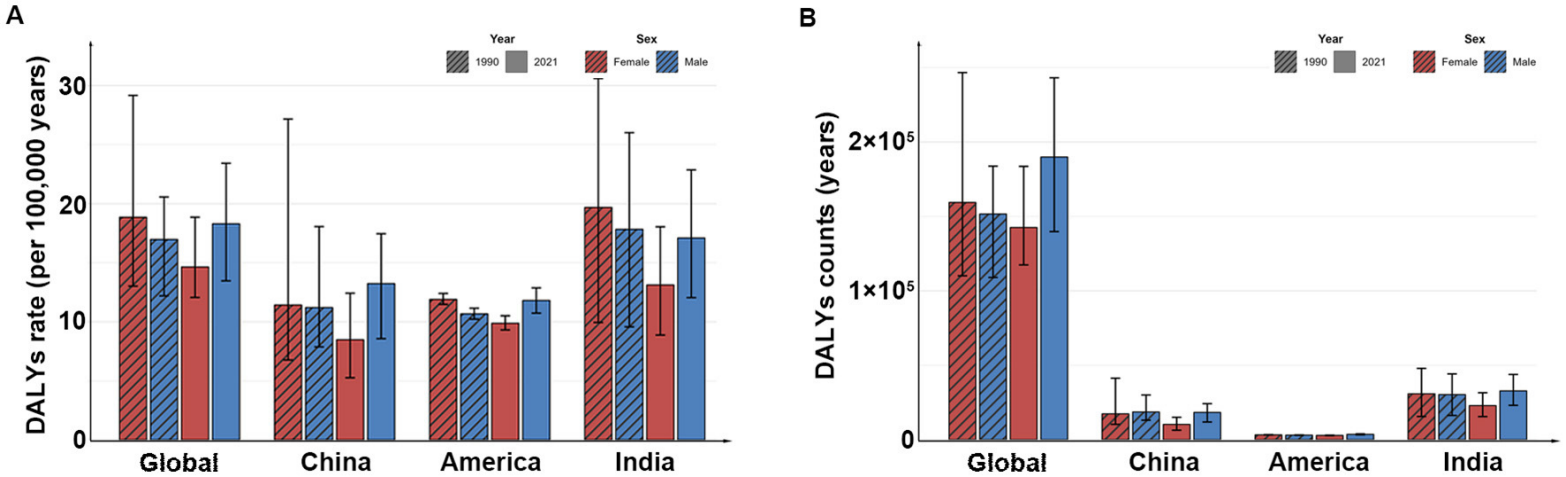
The DALYs rates **(A)** and the DALYs counts **(B)** in different countries in 1990 and 2021. DALYs, disability-adjusted life years.

In 2021, the total number of DALYs in the global child population due to MNBAC is about 332,534 (95% UI: 279,267–397,923), which is slightly increased compared with 311,078 (95% UI: 242,117–387,231) in 1990, while the DALYs rate has decreased compared with 1990, which is 17.89 per 100,000 years (95% UI: 13.92–22.27) in 1990 and 16.53 per 100,000 years (95% UI: 13.88–19.78) in 2021, with an average annual decline of 0.16% (95% CI: 0.09%−0.24%). Both in 1990 and 2021, the number and rate of DALYs in males were higher than those in females. At the national level, from 1990 to 2021, the number of DALYs in China and India has decreased, the number of DALYs in the United States has increased slightly, and the rate of DALYs in China, India and the United States has shown a slight downward trend, the most obvious decline is in India, which has decreased from 18.72 per 100,000 years (95% UI: 11.37–24.76) in 1990 to 15.2 per 100,000 years (95% UI: 11.76–19.36) in 2021. The average annual decline is 0.66% (95% CI: 0.51%−0.81%; Table 4, Figure 4).

**Table 4:** The DALYs rates and AAPCs of malignant neoplasm of bone and articular cartilage in childhood from 1990 to 2021.

**Characteristic**		**1990**		**2021**		**AAPC**
		**Counts**	**Rate (per 100,000 years)**	**Counts**	**Rate (per 100,000 years)**	
Global	Total	311,078 (242,117–387,231)	17.89 (13.92–22.27)	332,534 (279,267–397,923)	16.53 (13.88–19.78)	−0.16 (−0.24 to −0.09)
	Male	151,638 (109,004–183,792)	16.97 (12.2–20.57)	189,948 (139,829–243,152)	18.3 (13.47–23.42)	0.36 (0.31 to 0.41)
	Female	159,440 (110,114–246,661)	18.85 (13.02–29.17)	142,586 (117,433–183,631)	14.64 (12.06–18.86)	−0.74 (−0.85 to −0.63)
China	Total	36,007 (24,024–66,185)	11.31 (7.55–20.79)	28,611 (19,931–35,969)	11.02 (7.68–13.85)	0.09 (−0.55 to 0.73)
	Male	18,634 (13,100–30,036)	11.21 (7.88–18.06)	18,336 (11,877–24,190)	13.23 (8.57–17.46)	0.75 (0.13 to 1.37)
	Female	17,372 (10,294–41,311)	11.42 (6.77–27.16)	10,274 (6,373–15,039)	8.49 (5.26–12.42)	−0.8 (−1.51 to −0.09)
India	Total	61,121 (37,126–80,860)	18.72 (11.37–24.76)	55,696 (43,075–70,942)	15.2 (11.76–19.36)	−0.66 (−0.81 to −0.51)
	Male	30,301 (16,274–44,205)	17.83 (9.58–26.01)	32,779 (23,093–43,838)	17.1 (12.05–22.87)	0.15 (0 to 0.29)
	Female	30,820 (15,564–47,911)	19.68 (9.94–30.6)	22,917 (15,508–31,522)	13.12 (8.88–18.05)	−1.6 (−1.82 to −1.38)
United States	Total	6,302 (6,113–6,500)	11.27 (10.93–11.62)	6,455 (6,006–6,905)	10.86 (10.1–11.62)	−0.26 (−0.48 to −0.03)
	Male	3,056 (2,928–3,194)	10.67 (10.23–11.15)	3,585 (3,260–3,909)	11.81 (10.73–12.87)	−0.08 (−0.43 to 0.26)
	Female	3,246 (3,130–3,384)	11.9 (11.48–12.41)	2,870 (2,705–3,055)	9.87 (9.31–10.51)	−0.44 (−0.64 to −0.25)

AAPCs, average annual percent changes. DALYs, disability-adjusted life years.

## Discussion

Based on data from GBD 2021, this study comprehensively analyzes the disease burden and trends of childhood MNBAC in China, the United States, and India from 1990 to 2021, revealing the effectiveness and disparities in the prevention and control of childhood bone cancer across different countries and regions.

The results of the study indicate that the incidence and prevalence of childhood MNBAC have shown an upward trend globally over the past 30 years, while the mortality rate and DALYs rate have exhibited a downward trend. This trend reflects advancements in cancer treatment worldwide, particularly in the field of childhood oncology. However, it is noteworthy that despite the overall decline in mortality and DALYs rates, the global disease burden remains increasingly severe, with the number of new cases and prevalent cases of childhood MNBAC continuing to rise globally. Sarah et al. used the SEER database to describe the incidence and survival rate of osteosarcoma based on a total of 5,016 patients. The results showed that the incidence of osteosarcoma in patients under 10 years of age increased with age (13).

From a national perspective, as a developed and high-income country, the United States has the highest incidence and prevalence rates among the three countries. We believe this is related to its advanced medical level, more comprehensive cancer registration system, higher public health awareness, and greater investment in health. The increase in cancer incidence in high-income countries is attributed to the development of screening tests and early cancer monitoring in regions with effective medical systems, while children with cancer in middle- and low-income countries have limited access to diagnosis and treatment (14). The United States began establishing the Surveillance, Epidemiology, and End Results (SEER) database in 1974, which covered over 28% of the entire U.S. population from its inception (15). In contrast, China established its National Central Cancer Registry of China (NCCR) in 2002 (16). Meanwhile, India still lacks a unified and standardized national-level registration system, with cancer registrations primarily managed by regional hospitals or project-based initiatives. Furthermore, there are differences in the effectiveness of diagnosis and laboratory investigations between developed and less developed countries, which can affect data accuracy and the quality of cancer registration, leading to significant heterogeneity in cancer incidence rates among countries or regions with different levels of socioeconomic development (17, 18). In middle- and low-income countries, over 70% of children reside more than 1 h away from medical centers. These community-based health centers often lack sufficient staff and equipment to detect unusual pediatric issues, especially childhood bone tumors, resulting in delayed disease detection and referral (19). In resource-limited settings, limitations in access to healthcare and diagnostic capabilities for children with cancer lead to artificially low diagnosis rates.

Our study also found that the country with the highest mortality and DALYs rates for childhood MNBAC is India, while the country with the lowest rates is the United States. A well-trained team of pediatric orthopedic surgeons, anesthesiologists, oncologists, and nurses specializing in pediatric oncology plays a crucial role in the prognosis of childhood tumors. However, in low- and middle-income countries, there is a severe shortage of doctors in these pediatric subspecialties (18). When local control can be achieved through complete surgical resection of the tumor, surgeons may not have access to pediatric anesthesia and critical care, and subsequent delays may make complete resection of the tumor impossible, thereby reducing the chances of cure. Additionally, chemotherapy plays a vital role in the treatment of childhood MNBAC, such as osteosarcoma, and combination chemotherapy requires a reliable supply of multiple drugs simultaneously. In low- and middle-income countries, it may not be possible to purchase safe and effective anticancer drugs and maintain a reliable supply chain, making it difficult to achieve the desired treatment outcomes (18).

Furthermore, our study also found that the mortality rate of male children with MNBAC is slightly higher than that of female patients, both at the global and national levels, which is similar to the results of several previous studies. A study from the United States on Ewing's sarcoma reported that the 5-year survival rate for females is higher than that for males (20). Another study from Germany on osteosarcoma also indicated that female survival rates are higher than those for males, which may be related to poorer response to chemotherapy among male patients (21).

The trends we observed at both global and national levels represent the overall picture of this heterogeneous group of malignant neoplasms of bone and articular cartilage. Notably, major subtypes such as osteosarcoma and Ewing sarcoma exhibit significant differences in childhood incidence, age distribution, gender predilection, response to treatment, and prognosis. Since GBD 2021 data does not provide subtype disaggregation, our analysis cannot reveal the potentially distinct trajectories of burden changes across these important subtypes. Aggregated data may obscure the minimal contribution of specific subtypes (such as chondrosarcoma, which is extremely rare in childhood) to the overall metrics, and may also blur the distinct epidemiological patterns characteristic of the predominant subtypes (osteosarcoma and Ewin's sarcoma).

Based on the above findings, we recommend that globally, cancer registration systems should be strengthened, cancer registration systems should be improved, and data quality and completeness should be enhanced to provide a scientific basis for developing effective prevention and control strategies. Additionally, increased investment in medical resources is needed, particularly in low- and middle-income countries, where financial support for cancer treatment should be increased to improve the accessibility and quality of medical services. Early screening and intervention programs should be implemented for high-risk groups, such as male children, to increase early diagnosis rates and improve prognosis.

As the country with the largest population in the world, the Chinese government has made significant efforts over the past few decades to control the cancer burden, including the formulation of a series of policies such as “Healthy China 2030” and the “National Cancer Prevention and Control Program (2004–2010),” which provide professional guidance and support for cancer prevention in various regions, reflecting the government's emphasis on cancer prevention and control work and achieving remarkable results (22).

Although this study helps us understand the trend of the burden of MNBAC, it still has some limitations. Firstly, our data comes from GBD data, which uses data collected through mathematical models. However, in countries with different income levels, the accuracy of the estimates may vary. Secondly, despite the adoption of strict statistical methods, the differences in health information systems and reporting mechanisms in different regions may undermine the reliability of the results. Furthermore, MNBAC includes subtypes such as osteosarcoma, Ewing's sarcoma, and chondrosarcoma. However, the MNBAC data used in this study were aggregated and no further classification, statistical analysis, or discussion of specific tumors was conducted. In view of these limitations, the results of this study should be interpreted with caution.

## Conclusion

In summary, malignant bone and articular cartilage tumors in children remain a significant public health challenge globally. By strengthening monitoring, increasing investment in medical resources, promoting early screening and intervention, and enhancing international cooperation and exchange, we hope to further reduce the burden of childhood bone cancer and improve children's quality of life and health standards.

## Data Availability

Publicly available datasets were analyzed in this study. This data can be found at: https://www.healthdata.org/data-tools-practices/interactive-visuals/gbd-results.
